# Diagnostic accuracy of ultrasonography compared to unenhanced CT for stone and obstruction in patients with renal failure

**DOI:** 10.1186/1471-2342-4-2

**Published:** 2004-07-29

**Authors:** M Hammad Ather, Aftab H Jafri, M Nasir Sulaiman

**Affiliations:** 1 Section of Urology, Department of Surgery, Aga Khan University, Karachi, Pakistan

## Abstract

**Background:**

To determine accuracy of ultrasound (US) kidney, ureter and bladder (KUB) compared to un-enhanced helical CT (UHCT) in patients with renal failure in the diagnosis of stone and obstruction.

**Methods:**

This is a case controlled study conducted in the period from June 2000 to July 2003 at a university hospital. All patients had both US and UHCT scan. Patients with serum creatinine ≥ 1.8 mg/dl were included in the study. Only direct visualization of stone was considered as confirmatory. In both the studies, UHCT and US, presence of stone and obstruction were noted. The relevant biochemicals, radiological and clinical records of all the patients were analyzed. Data was analyzed using commercially available software.

**Results:**

During the period of study 864 patients had UHCT for evaluation of the urinary tract in patients presenting with flank pain. Out of these 34 patients had both UHCT and US done within a span of one day and had serum creatinine of ≥1.8 mg/dl. Mean age was 48 ±15.8 years and 59% of patients were males. UHCT identified renal stones in 21 (62%), whereas 17 of these were identified on US, with a sensitivity of 81%. Of the four patients with renal stones missed on US, three were identified on plain x-ray; the mean size of stones missed was 6.3 mm. Of the 22 (65%) patients with ureteric stone on UHCT, US could only identify 10; a further 7 were identified on x-ray KUB, giving a sensitivity of 45% (US alone) and 77% (US with x-ray KUB).

**Conclusions:**

US is sensitive and specific for renal stones, 81% and 100% and for hydronephrosis, 93% and 100%, respectively. Its sensitivity to pick ureteric stone (46%) and to identify hydroureter (50%) is low. Addition of x-ray KUB abdomen increases the sensitivity for ureteric stones to 77%.

## Background

Intravenous urography (IVU) has been the gold standard for the radiological survey of intra renal collecting system, ureter and bladder. Choice of imaging for urinary tract in patients with raised serum creatinine is limited to non-contrast enhanced studies. These considerations have led to the use of other modalities like combination of plain abdominal radiography (KUB) and gray scale ultrasound (US) kidney, ureter and bladder. More recently use of non-contrast enhanced CT (UHCT) and magnetic resonance urography (MRU) in the evaluation of flank pain has received increasing attention [1,2]. Work in the past decade has shown UHCT to be highly sensitive and specific [1,3,4]. It is highly sensitive for both renal and ureteric stone [3]. The probability of misdiagnosis in distal ureter with multiple phleboliths is still a significant problem. Presences of tissue rim [3,5] and comet tail [5] signs along with secondary signs of obstruction are helpful in these situations.

Ultrasound has many inherent advantages, which includes lack of radiation, universal availability, in expensive and non-invasive. It is useful in the diagnosis of renal and ureteric calculi. Stones on US are characteristically demonstrated as highly echogenic foci with distinct acoustic shadowing. The greatest challenge with regard to US is the identification of ureteral calculi, particularly in it's abdominal, and upper pelvic course. This limitation of US is due to its inability to scan retroperitoneum due to overlying bowel loop, and bony structures [4,6]

Plain abdominal radiograph also lacks specificity, as phleboliths are not readily differentiated from ureteric calculi. Plain radiographs are also not sensitive to radiolucent calculi and non-calculus obstruction.

In the present study we have compared the diagnostic accuracy of UHCT with US with x-ray KUB for the diagnosis of renal and ureteric stones in patients with raised serum creatinine precluding the use of contrast enhanced study.

## Methods

This is a case controlled study conducted in the period from June 2000 to July 2003 at a university hospital. All patients who had both US and un-enhanced helical CT (UHCT) scans performed within a span of 24 hours and a serum creatinine ≥ 1.8 mg/dl were included in the study. Serum creatinine of 1.8 mg/dl is considered as a cut off for use of intravenous contrast by our radiology department.

The radiologist's reports on CT, CT films and medical records of patients for suspected renal/ureteral colic were reviewed. The UHCT were obtained on a Cti/pro single slice helical CT scanner (General Electrical medical systems, Milwaukee, WI). The exposure factors setting were KVp 130 and mAS 200–250. All scans were obtained from the upper border of T12 vertebral body to the lower border of symphysis pubis using 5–7 mm collimation, without the use of oral or intravenous contrast material. Patients were placed in supine position with full urinary bladder at the time of the UHCT. Additional prone films were taken whenever the radiologist needed a better description of suspected distal ureteric calculi. Ultrasound KUB was done using 3.75 MHz surface probe. All ultrasounds were seen and reported after being reviewed by a senior radiologist. Secondary signs of obstruction, like hydronephrosis, hydroureter, nephromegaly, perinephric and periureteric stranding were also noted but only direct visualization of stone was considered confirmatory.

The relevant biochemicals, radiological and clinical records of all the patients were analyzed. In the studies, UHCT, and US presence of stone and obstruction were noted. Data was analyzed using commercially available software (statistical package for social sciences version 8.0).

## Results

During the 38-month period of study 864 patients had UHCT for evaluation of the urinary tract in patients presenting with flank pain. Out of these 34 patients had both UHCT and US done within a span of one day and had serum creatinine of ≥ 1.8 mg/dl. UHCT was considered as a reference point in the study as all stones identified on the CT were subsequently reconfirmed with interventional treatment or history of spontaneous passage.

Mean age was 48 ± 15.8 years (range 20–76 years), 59% of patients were males. UHCT identified renal stones in 21 and ureteric stones in 22 patients. Forty-two (98%) of these stones were confirmed clinically (history of spontaneous passage), or during treatment with ureteroscopy, percutaneous nephrolithotomy and extracorporeal shock wave lithotripsy. Of the 21 renal stones, only 17 were identified on US, with a sensitivity of 81%, specificity and positive predictive value of 100% and negative predictive value of 77%. Of the four patients with renal stones missed on US, three were identified on x-ray KUB; the mean stone size of stones missed on US was 6.3 mm. In all cases US and x-ray KUB were performed prior to the UHCT.

Of the 22 patients with ureteric stone, on UHCT, US could only identify 10. Twelve patients with ureteric stones identified on UHCT were missed on US. The mean size of stones missed was 6.1 mm (range 3–15 mm). The sensitivity, specificity, positive and negative predictive values were 46, 100, 100 and 50% respectively. A further 7 patients, missed on US, were identified on x-ray KUB. The overall sensitivity of US and x-ray KUB was 77%. The impact of location of stones missed on US is shown in table 1 and 2.

**Table 1:** The impact of location on detection of stone and hydronephrosis by UHCT and US.

	**Upper ureter**	**Middle ureter**	**Distal ureter**
**n**	6	14	2
**Identified on CT**	6	14	2
**Identified on US**	4	5	1
**Hydrouretero-nephrosis on CT**	6	14	2
**Hydrouretero-nephrosis on US**	4	8	1

CT un enhanced helical CT US gray scale ultrasound

**Table 2:** Site and size of stones missed on US, the mean size of stones missed was 6.1 mm.

	**Stone identified/total**	**Mean size**
**Upper ureter**	2/6 (33%)	6 mm
**Middle ureter**	9/14 (64%)	5 mm
**Distal ureter**	1/2 (50%)	7 mm

## Discussion

IVU has been the traditional imaging modality of choice for evaluation of patients suspected of having urolithiasis and obstruction. Choice of imaging for urinary tract in patients with renal insufficiency and renal failure is limited to non-contrast enhanced studies. Gray scale ultra sonography is the most effective way to exclude sub acute or chronic obstruction. However, regular gray scale US is not accurate in minimally dilated obstruction, such as with partially obstructing ureteric stone; in one series, 4–5% of patients with obstruction showed minimal or no upper tract dilatation [7]. Duplex Doppler is less effective in acute and incomplete obstruction since obstruction for longer than six hours is necessary to show a consistently elevated resistive index (RI) [8]. Therefore, we did not evaluate RI values or ureteric jets in our study. Others have also recently examined the role of RI with disappointing results. Cronan showed that the addition of RI did not improve the 77% sensitivity of gray scale US in that series [9].

US has high sensitivity for renal stones and presence of hydronephrosis. But its sensitivity for ureteral calculi is low. In one study, where IVU was compared with US, the sensitivity of US for ureteral calculi was only 37% (direct visualization) and when hydronephrosis was included as positive sign for ureteral calculi the sensitivity increased to 74% [10].

**Figure 1:**
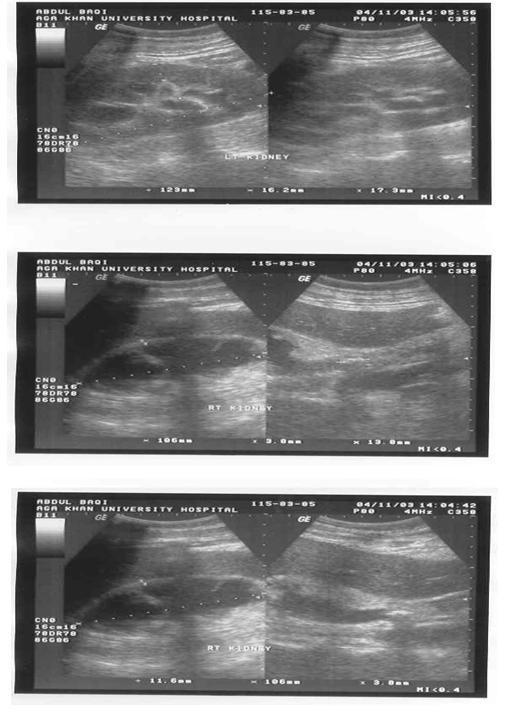
US of 65 years old male presented to emergency room with bilateral flank pain, nausea and vomiting for the past 1 week. He had an ultrasound in a peripheral hospital, which identified hydronephrosis on the right side, and percutaneous nephrostomy tube was placed. His left kidney showed hydronephrosis with renal stone (upper picture). This scan shows small-scarred right kidney (middle picture), pigtail catheter could be identified (arrow) and a proximal ureteric stone could also be seen (lower picture).

**Figure 2:**
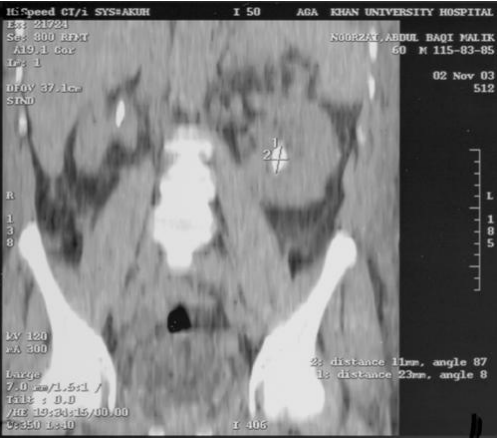
Reformatted unenhanced helical CT image of the same patient showing small-scared right kidney with proximal ureteric calculus and hydroureter. Left kidney shows hydronephrosis and renal calculus.

Recent studies have demonstrated that UHCT is an excellent method for demonstrating urolithiasis and obstruction in patients presenting with flank pain [1-3]. Smith et al [3] showed UHCT to be more effective than IVU in identifying ureteral stones. In another comparative study, Sommer et al [4] noted that reformatted (see Figure 2), UHCT images are superior to US and plain radiographs. Data from our institution showed that UHCT has a sensitivity of 99% and specificity of 98% in the diagnosis of ureteric calculi [1](US and plain radiograph Figure 1 and Figure 2). Additionally UHCT could also suggest additional, non-urinary tract abnormalities as cause of flank pain in 12% of patients [11].

Sensitivity of US is reported to be 96 % for renal stones and is 100% sensitive for stones larger than 5 mm in reported literature [1,12]. In our study US had sensitivity and negative predictive value of 81 and 77%. If x-ray KUB is added the sensitivity increased to 95%. The four patients with renal stones missed on US had a mean stone size of 6.3 mm. Lower sensitivity in our work could be due to small sample size.

In the present study US alone had a sensitivity of only 46% for direct visualization of ureteric stones, in combination with x-ray KUB it increased to 77%. The 12 stones missed on US had a mean size of 6.1 mm (range 3–15 mm). Majority of stones missed on US were in the middle ureter (n = 9), 2 were in the proximal ureter and one in the distal ureter. X-ray KUB identified 7 of the 12 stones missed on US. Of these 12 patients with ureteric stones missed only 2 had hydroureter. Presence of hydroureter in patients with ureterolithiasis is valuable as it allows the ureter to be traced to the level of obstruction. Majority (9 out of 12) of stones missed on US were in the middle ureter, an area often obscured by bowel gas.

## Conclusions

In summary, US is the first imaging study for evaluating the patients with previously undiagnosed renal failure. It helps the clinician to separate end stage renal disease from potentially reversible obstructive uropathy secondary to urolithiasis. US is highly sensitive and specific for renal stones in patients with renal failure, it lacks sensitivity for ureteric calculi particularly when they are in the middle ureter. Even addition of x-ray KUB to US misses about a quarter of ureteric stones; we therefore recommend using UHCT if ureterolithiasis is clinically suspected or US and x-ray KUB examinations are equivocal. Due to small sample size, findings of this study should be validated by other studies on a larger cohort of patients.

## Competing interest

None declared.

## Authors' contribution

MHA conceived the idea, analyzed data and drafted the manuscript.

AHJ collected the data and analyzed the results.

MNS analyzed results and drafted the manuscript.

## Pre-publication history

The pre-publication history for this paper can be accessed here:


